# Tillage and herbicide reduction mitigate the gap between conventional and organic farming effects on foraging activity of insectivorous bats

**DOI:** 10.1002/ece3.3688

**Published:** 2017-12-30

**Authors:** Kévin Barré, Isabelle Le Viol, Romain Julliard, François Chiron, Christian Kerbiriou

**Affiliations:** ^1^ Centre d'Ecologie et des Sciences de la Conservation Muséum national d'Histoire naturelle, UMR 7204 MNHN‐CNRS‐UPMC Paris France; ^2^ Agrosolutions Paris France; ^3^ Station de biologie marine Muséum National d'Histoire Naturelle Place de la croix Concarneau France; ^4^ Ecologie Systématique Evolution, AgroParisTech CNRS, Université Paris‐Sud, Université Paris‐Saclay Orsay France

**Keywords:** chiroptera, farming practices, farmland biodiversity, pesticides, plowing, weed control

## Abstract

The increased use of pesticides and tillage intensification is known to negatively affect biodiversity. Changes in these agricultural practices such as herbicide and tillage reduction have variable effects among taxa, especially at the top of the trophic network including insectivorous bats. Very few studies compared the effects of agricultural practices on such taxa, and overall, only as a comparison of conventional versus organic farming without accurately accounting for underlying practices, especially in conventional where many alternatives exist. Divergent results founded in these previous studies could be driven by this lack of clarification about some unconsidered practices inside both conventional and organic systems. We simultaneously compared, over whole nights, bat activity on contiguous wheat fields of one organic and three conventional farming systems located in an intensive agricultural landscape. The studied organic fields (OT) used tillage (i.e., inversion of soil) without chemical inputs. In studied conventional fields, differences consisted of the following: tillage using few herbicides (T), conservation tillage (i.e., no inversion of soil) using few herbicides (CT), and conservation tillage using more herbicide (CTH), to control weeds. Using 64 recording sites (OT = 12; T = 21; CT = 13; CTH = 18), we sampled several sites per system placed inside the fields each night. We showed that bat activity was always higher in OT than in T systems for two (*Pipistrellus kuhlii* and *Pipistrellus pipistrellus*) of three species and for one (*Pipistrellus* spp.) of two genera, as well as greater species richness. The same results were found for the CT versus T system comparison. CTH system showed higher activity than T for only one genus (*Pipistrellus* spp.). We did not detect any differences between OT and CT systems, and CT showed higher activity than CTH system for only one species (*Pipistrellus kuhlii*). Activity in OT of *Pipistrellus* spp. was overall 3.6 and 9.3 times higher than CTH and T systems, respectively, and 6.9 times higher in CT than T systems. Our results highlight an important benefit of organic farming and contrasted effects in conventional farming. That there were no differences detected between the organic and one conventional system is a major result. This demonstrates that even if organic farming is presently difficult to implement and requires a change of economic context for farmers, considerable and easy improvements in conventional farming are attainable, while maintaining yields and approaching the ecological benefits of organic methods.

## 1 INTRODUCTION

Halting the loss of biodiversity, recognized as a crucial aim for humanity (Cardinale et al., 2012), has resulted in the adoption of different environmental policies to reduce the impact of anthropogenic changes. As land‐use changes and agriculture intensifies, farming, particularly crop farming, is a major driver of biodiversity loss (Maxwell, Fuller, Brooks, & Watson, 2016). There is an urgent need to reconcile nature conservation and agricultural production on large spatial scales. Two contrasting scenarios optimizing nature conservation and production have so far been proposed and widely discussed: integrate conservation and production functions in heterogeneous landscapes (land sharing) or separate farming activities from nature conservation in homogeneous landscapes (land sparing; Fischer et al., 2008). Both strategies are controversial, their effectiveness is clearly dependent on conservation aims (species vs. ecosystem functions), land‐use intensity, and landscape‐context (Kleijn, Rundlöf, Scheper, Smith, & Tscharntke, 2011). None appear satisfactory in large productive agricultural regions where landscapes have been heavily modified, where production has a strong economic stake, but where conservation of species and ecosystem functions and services are crucial (Power, 2010), especially as these systems cover large areas on a global scale (agriculture area: 38.5%; FAO, 2011). In such intensive regions, the best complementary and conciliatory approaches in the short term (i.e., without big changes in the political choices of production) are likely to be those that increase biodiversity potential (species, abundance) without reducing production and the surface of seminatural habitats, a so‐called win‐no loss situation (Teillard, Doyen, Dross, Jiguet, & Tichit, 2016). This could be achieved by improving farming systems through changes in practice, from those that are the most negative for biodiversity to those that are both the least negative and not antagonistic to production (Petit et al., 2015). The threat from crop farming is not only a product of land‐use changes including clearing for cultivation, homogenization of the agricultural landscape, and fragmentation of associated habitats such as woodlands (Benton, Vickery, & Wilson, 2003), but also the intensification of practices within croplands such as increased use of fertilizers, pesticides, the simplification of crop rotation (Bengtsson, Ahnström, & Weibull, 2005; Benton et al., 2003), and the replacement of genetically diverse traditional varieties by homogeneous modern varieties (Hoisington et al., 1999).

With the aim of mitigating biodiversity loss in agricultural landscapes, environmental policies have been launched and regularly reformed in numerous countries, such as across Europe with the Common Agricultural Policy. However, although there are possibilities for improvement, previous reforms do not appear to be satisfactory for biodiversity conservation (Pe'er et al., 2014). For the environmental element, reforms consist of Agri‐Environment Schemes (AES) that are based on offering financial incentives to farmers to implement and protect areas and lines of vegetation, using fewer agrochemicals, or employing alternative pasture methods (Kleijn & Sutherland, 2003). Agri‐Environment Schemes occurred in 22% of farms during the period 2000–2009 (Zimmermann & Britz, 2016) and 21% of the Utilized Agricultural Area (Eurostat, 2009). Such schemes have had so far marginal to moderately positive effects on biodiversity (Kleijn et al., 2006); even if AES can have substantial effects at a local scale, they are not halting national declines in populations (Gamero et al., 2017). In addition, although some key elements have been identified on a larger scale than AES as affecting local farmland biodiversity such as landscape and habitat heterogeneity and connectivity (Benton et al., 2003; Tscharntke, Klein, Kruess, Steffan‐Dewenter, & Thies, 2005), some elements of local management are also known to be highly positive for biodiversity, such as crop diversification (Gurr et al., 2016) and farming practices (Hole et al., 2005).

The intensification of pesticide use is known to negatively affect many taxa on a large scale (Geiger et al., 2010) as well as tillage (i.e., inversion of soil; Holland, 2004) and the shortening of crop rotations (Dick, 1992; Hole et al., 2005). However, agricultural practices that aim to conserve biodiversity within agricultural landscapes such as restrictions on chemical inputs in organic farming have variable efficiency among taxa (Fuller et al., 2005). In addition, due to the multiplicity of ways to control weeds leading to a trade‐off between herbicide use and tillage intensity, opposite effects of conservation tillage on biodiversity have also been observed (i.e., no inversion of soil; Filippi‐Codaccioni, Clobert, & Julliard, 2009; Flickinger & Pendleton, 1994; Lokemoen & Beiser, 1997; Shutler, Mullie, & Clark, 2000). Conservation tillage is a common practice used in 28.4% of the total arable land of France (Agreste, 2011), often with an economic aim when the fuel needed for tillage meant it was less profitable than a conservation tillage using more herbicides to control weeds. Such uncertainties about practices necessitate an accurate study of a simultaneous gradient of farming practices within the same landscape. In theory, such a gradient should be studied in systems with similar yields to calculate the relative effects of tillage intensity and chemical inputs in order to define optimal farming practices that respond to both the aims of production and biodiversity conservation. However, studies on the relationship between farming practices and biodiversity are lacking for several taxa, and/or results are frequently controversial, indeed some farming practices such as conservation tillage include several distinct and more or less intensive methods that so far have not often been taken into account. Focusing on the response of species, at the top of trophic network, such as bats that are abundant, strictly insectivorous and considered as effective bioindicators (Park, 2015; Russo & Jones, 2015), may thus be helpful for better assessing the effect of such farming practices. Insectivorous bats also provide many ecosystem services (Kunz, de Torrez, Bauer, Lobova, & Fleming, 2011) such as a huge economic advantage in agriculture with a gain of billions of dollars each year (Boyles, Cryan, McCracken, & Kunz, 2011). Several species identified as threatened by the International Union for Conservation of Nature are largely affected by intensive agriculture (Azam, Le Viol, Julien, Bas, & Kerbiriou, 2016). Moreover, AES do not seem to encourage conservation of such species in conventional farming (Fuentes‐Montemayor, Goulson, & Park, 2011; MacDonald et al., 2012; Park, 2015) even having negative effects sometimes (Fuentes‐Montemayor et al., 2011) that accentuate the urgent need to assess the effects of farming practices.

To our knowledge, only very few studies have compared conventional with organic farming, and only in a general way with no gradient of practices in both systems. Moreover, results diverged according to studies, showing significantly positive effects of organic farming compared to conventional farming on activity (Fuller et al., 2005; Wickramasinghe, Harris, Jones, & Jennings, 2004; Wickramasinghe, Harris, Jones, & Vaughan, 2003), species richness, and diversity (Fuller et al., 2005). It was also found that there were no differences (Pocock & Jennings, 2008) but driven differences in boundaries between organic and conventional, as well as two cases of significant negative effect on diversity (Fuller et al., 2005) and activity (MacDonald et al., 2012). In all these studies, the activity of strictly insectivorous bats was measured during their foraging activity, known to be driven by arthropod availability (Charbonnier, Barbaro, Theillout, & Jactel, 2014; Hayes, 1997). Thus, the lack of consensus about effects of farming systems on bats could be linked to an effect of the gradient of practices on their arthropod prey, such as herbicides and tillage intensity (Evans, Shaw, & Rypstra, 2010; Pereira et al., 2007; Rodríguez, Fernández‐Anero, Ruiz, & Campos, 2006; Taylor, Maxwell, & Boik, 2006). Although some studies revealed herbicide accumulation in bats along the farming intensification gradient (Bayat, Geiser, Kristiansen, & Wilson, 2014; Stechert, Kolb, Bahadir, Djossa, & Fahr, 2014), the relative effects of herbicide use and tillage intensity on crop attractiveness for bats remain unstudied.

The aim of this study is to analyze the potential role of farming practices in providing ecological benefits for bats, and the possibilities of conventional farming to draw closer to the beneficial effects of organic farming. Specifically, we compare bat foraging activity simultaneously on wheat crops of one organic and three conventional farming systems located in a narrow intensive agricultural landscape, setting us free from the landscape effect known to be greater than the practice effect (Bengtsson et al., 2005). This allows the analysis of the effect of tillage and herbicide intensification on bat activity using a comparison of the following systems:

1. Organic tillage fields (OT, organic farming): inversion of soil without herbicides
2. Conservation tillage fields (CT, conventional farming): no inversion of soil using two herbicide passes
3. Conservation tillage fields using more herbicide (CTH, conventional farming): no inversion of soil using three herbicide passes
4. Tillage fields (T, conventional farming): inversion of soil using two herbicide passes

We expected that organic tillage would have a positive effect on bat activity and species richness compared to the three other conventional systems, thanks to the lack of herbicides, such as previously shown on a large scale by Wickramasinghe et al. (2003). We also hypothesize that tillage and herbicide reductions in conventional systems could help to mitigate this gap, influencing availability of arthropod prey known to drive bat activity.

## 2 METHODS

### 2.1 Agricultural context of the study area

The study was conducted in the Île‐de‐France region, France, in an intensive agricultural landscape among some of the main productive areas in Europe (Table S1.1). This region is covered by 59% of agricultural areas similar to that found at a national level (Table S1.2), dominated by arable land (90%) for intensive cereal crops (62% of wheat and barley), rape (14%), corn (14%), sugar beet (6%), and peas (4%; Agreste, 2010). Organic farming represents 2.5% of the utilized arable land (UAL) on a national level, and, respectively, 1.4% and 4.1% in the region and in the study area, with a positive trend in France (+52.7%) over the 2011–2015 period (Table S1.3). At the opposite end of the spectrum, pesticide use has increased by 5.8% since 2011 at the national scale (Ecophyto, 2015); only herbicides showed a significant positive trend for the period of time 2008–2013 (Hossard, Guichard, Pelosi, & Makowski, 2017). Similarly, conservation tillage takes up 28.4% of the UAL in France, but only 21.4% and 14.3% in the region and in the study area, respectively (Table S1.4).

### 2.2 Sampling design

**Figure 1:**
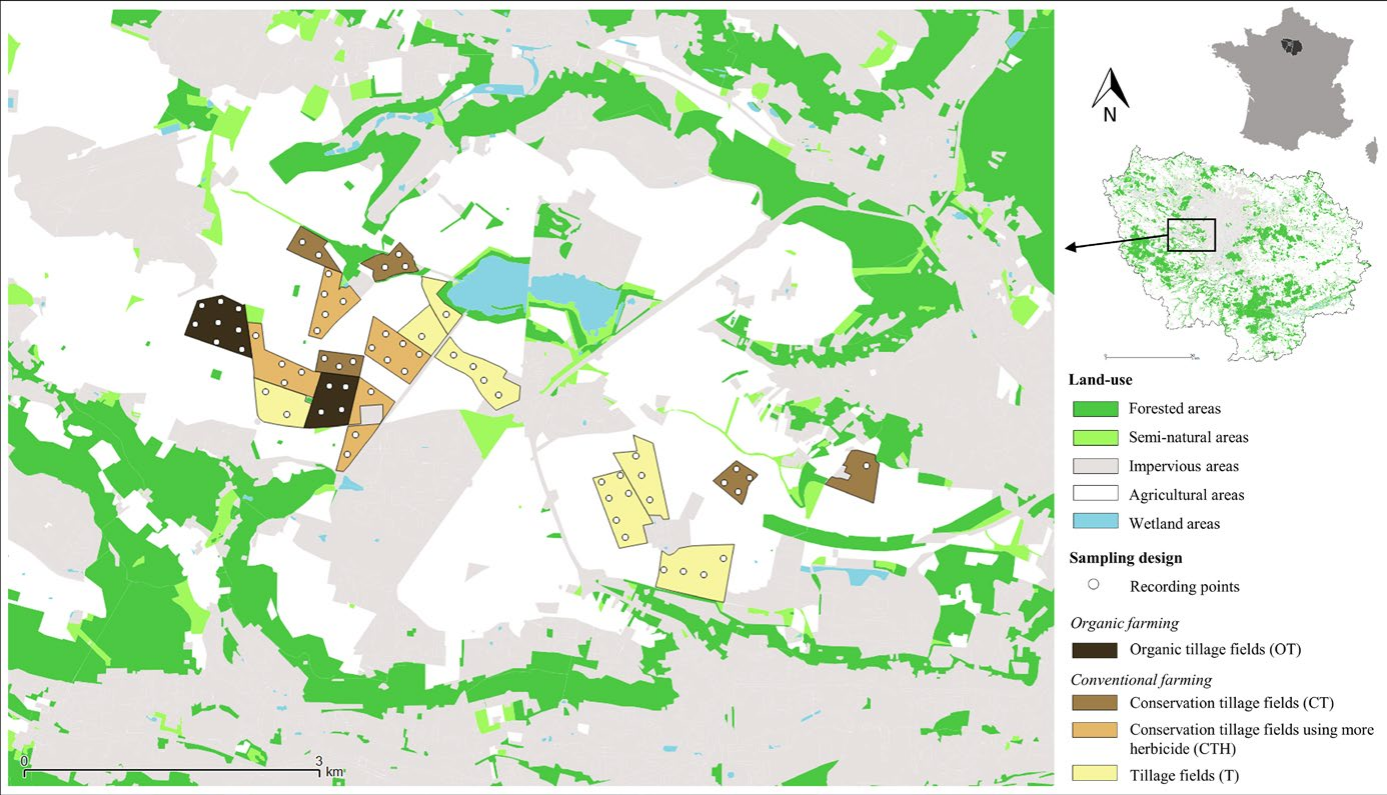
Land‐use map of the study area showing sampling sites inside wheat fields of the four studied farming systems (OT, organic tillage fields; CT, conservation tillage fields; CTH, conservation tillage fields using more herbicide; T, tillage fields)

We compared bat activity across one organic and three conventional farming systems through recordings of bat activity on wheat crops in all four farming systems simultaneously. We sampled 64 sites: 12 sites for organic tillage fields (OT), 13 for conservation tillage fields (CT), 18 for conservation tillage fields using more herbicide (CTH), and 21 for tillage fields (T), distributed inside 19 contiguous fields over a 3.5 km radius on the same agricultural plateau (Figure 1).

Thanks to the contiguity of fields and the small spatial scale, landscape and soil composition are very similar among the four systems. This allowed to avoid variance source connected with landscape and to study in detail the choice of plot of a given system rather than another by individuals. In addition, sampling sites inside the fields were chosen in order to limit differences in surrounding land‐use in the studied farming systems (Figure S2.1).

### 2.3 Features of farming practices studied

Farming systems differed in their method of weed control in wheat crops. During the intercrop period, between July (harvest of the cash crop) and October (sowing of the new crop), all four systems used one to two harrowings in August; OT and T used a tillage (i.e., inversion of soil to 30‐cm depth) followed by a smoothing to prepare the seedbed; CT and CTH used conservation tillage (i.e., no inversion of soil) using, respectively, a single decompaction (superficial tillage to lighten the soil without destructuring) and a decompaction associated with one herbicide. After sowing, during the growing period, the three conventional systems (T, CT, and CTH) applied one herbicide just after sowing, as well as in March, followed by one fungicide in April and May, and then two in June. No insecticide was applied. Active molecules of herbicides and fungicides used were the same for the three conventional systems (Tables S3.1 and S3.2). The OT system did not use chemical pesticides, just a mechanical weed control using a hoeing machine in March, April, and June (Table 1).

**Table 1:** Chronology of interventions on studied wheat fields over an entire year in four farming systems. Bat monitoring was performed in June

Farming systems	Chronology of interventions							
	July	August	September	October	March	April	May	June
**Organic farming**								
Organic tillage (OT)	Harvest	Harrowing	Tillage + smoothing	Sowing	Mechanical weed	Mechanical weed	Ø	Mechanical weed
**Conventional farming**								
Conservation tillage (CT)	Harvest	Harrowing	Decompaction	Sowing + herbicide (x1)	Herbicide (x1)	Fungicide (x1)	Fungicide (x1)	Fungicide (x2)
Conservation tillage using more herbicide (CTH)	Harvest	Harrowing	Decompaction + Herbicide (x1)	Sowing + herbicide (x1)	Herbicide (x1)	Fungicide (x1)	Fungicide (x1)	Fungicide (x2)
Tillage (T)	Harvest	Harrowing	Tillage + smoothing	Sowing + herbicide (x1)	Herbicide (x1)	Fungicide (x1)	Fungicide (x1)	Fungicide (x2)

In all systems, wheat crops were sown every two years (for more details on crop type between wheat crops see Table S3.3). Organic fields were established for over ten years; conventional and conservation tillage were performed on different fields each year.

### 2.4 Bat monitoring

Recordings were carried out from the 16th to the 23rd of June 2016 in the seasonal peak of bat activity as recommended by the French national bat‐monitoring program “Vigie‐Chiro” (http://vigienature.mnhn.fr/), under favorable weather conditions without rain, with low wind speeds (<7 m/s), and temperatures higher than 12°C.

In order to optimize and standardize comparisons, we sampled simultaneously using continuous acoustic monitoring, one to four sites for each farming system (OT/CT/CTH/T) on the same night (a total of six to eleven sites per night; Table S4.1) from 30 min before sunset to 30 min after sunrise (Skalak, Sherwin, & Brigham, 2012). The bat taxa are known for substantial internight, seasonal, and year variations in abundances (Hayes, 1997). Thus, a sampling design focused within a short period, where several sites were simultaneously sampled for each farming system on the same night, allowed to minimize this temporal source of variance. This design also allowed to control for these potential variations using nested models performed on date (see Section 2.5). In addition, the eight sampled nights were performed during the lactation period, a season with high energetic constraints, allowing to study a more accurate foraging selection.

Standardized echolocation calls were recorded using one SM2BAT detector per site. All simultaneously sampled sites were separated by at least 300 m to avoid simultaneous recordings. In addition, all sites were always placed inside the field at a minimum of 40 m distance from the boundary to minimize its effects. The detectors automatically recorded all ultrasounds (>12 KHz) while maintaining the characteristics of the original signals. In addition, note that the bat activity we used is not an abundance, but a metric more sensitive to habitat quality: for example, one bat foraging all night within a sampled site will produce a huge value of bat activity instead of some bats crossing the sampled site.

For the first step, echolocation calls were detected and classified to the most accurate taxonomic level using the TADARIDA software (Bas, Bas, & Julien, 2017) which allows a confidence index to be assigned to each classification of call. For the second step, all echolocation calls were checked using BatSound© software except the most represented species, *Pipistrellus pipistrellus*, for which we only checked 20% from the 0.5 confidence index due to a high quantity of calls (see Table S4.2 for more details on the identification procedure). In addition to the calls assigned to *P. pipistrellus*,* P. kuhlii*, and *P. nathusii*, we constructed three groups (*Nyctalus* spp, *Plecotus* spp, and *Myotis* spp) as contact with these taxa were associated with low occurrence, or difficulty in identification (Obrist, Boesch, & Fluckiger, 2004).

As it is impossible to determine the number of individual bats from their echolocation calls, we calculated a bat activity metric (bat passes), calculated as the number of bat passes per night per species, where a bat pass is defined as a single or several echolocation calls during a five‐second interval.

### 2.5 Statistical analysis

We performed general linear mixed models (GLMM, R package *lme4*) using bat activity (number of bat passes of species and genus) and species richness as response variables associated to a negative binomial error distribution (Zuur, Ieno, Walker, Saveliev, & Smith, 2009), except for the *Nyctalus* spp. genus for which a binomial error distribution was used due to excessively low variation in abundance. Note that occurrences were too low for *Plecotus* spp. and *Myotis* spp. (present in <10% of the 64 sites) to perform models, but these genera were used in species richness. We tested the type of farming system in models as fixed effects (composed of four factors: OT, CT, CTH, and T), and we included scaled landscape covariates (distance to wetlands, forests, hedgerows, roads, boundaries, urban areas) known as good predictors of bat activity for the species studied (Boughey, Lake, Haysom, & Dolman, 2011; Lacoeuilhe, Machon, Julien, & Kerbiriou, 2016).

**Figure 2:**
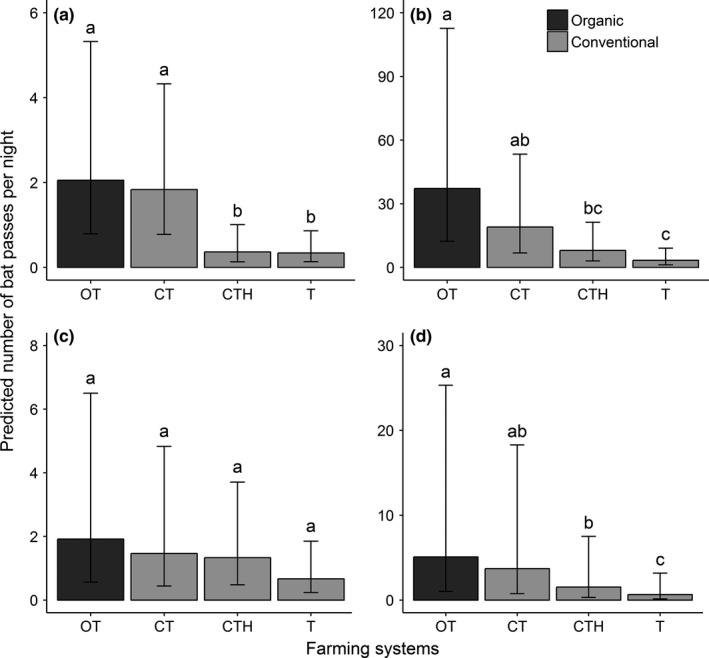
Predicted number of bat passes per night and the associated 95% confidence intervals under the 4 systems (see Table 1 for description) across organic and conventional farming for (a) *Pipistrellus kuhlii*, (b) *P. pipistrellus*, (c) *P. nathusii* and (d) *Pipistrellus* spp., The a, b and c letters shared between two or more systems refer to no significant differences

To avoid overparameterization due to a limited dataset (species, *Nyctalus* spp. genus and richness: *n* = 64; *Pipistrellus* spp. genus including the three *Pipistrellus* species: *n* = 192), we chose to build models including six degrees of freedom (*df*). We performed a hierarchical partitioning (R package *hier.part*) to identify the first three covariates (3 *df*) having the best conjoint contributions, in order to implement them with the farming system variable (3 *df*) in full models (Table S4.3). According to the sampling design (i.e., simultaneous recordings of bat activity among four farming systems on the same night), we included the date in the models as a random effect with the aim being to check for internight variations. For the *Pipistrellus* spp. model, we added a second random effect on the three species composing the genus, in order to take into account activity variations among species. From full models, we checked potential multicollinearity problems using two successive approaches. In a first step, we tested differences in covariates between farming systems (Kruskal Wallis tests; Table S4.4), and checked correlations between covariates (Table S4.5). We detected two significant differences between farming systems among the six landscape variables (i.e., the distance to hedgerows and the distance to wetlands; Figures S2.1 and S4.1), as well as correlations between distances to boundaries and roads and between distances to wetlands and hedgerows (Table S4.5). To take into account these correlated covariates, we did not simultaneously include them in the modeling procedure. In a second step, we checked there were no multicollinearity problems performing variance inflation factors (VIF) using the *corvif* function (R package *AED*; Zuur, Ieno, & Elphick, 2010) on each full model. All variables showed a VIF value <2, meaning there was no striking evidence of multicollinearity (Chatterjee & Hadi, 2006). We generated, based on full models (Table S4.3), a set of candidate models containing all possible variable combinations ranked by corrected Akaike Information Criterion (AICc) using the *dredge* function, but not simultaneously including correlated covariates. For each set of candidate models, we did multimodel inference averaging on a delta AICc <2 using the *model.avg* function to obtain an averaged regression coefficient for each fixed effect (R package *MuMIn*; Barton, 2015; Table S4.6). We used the *allEffects* function (R package *effects*) to get a predicted activity of bat species from the best models in Figure 2. We did not detect spatial autocorrelation on residuals of each best model using *dnearneigh* and *sp.correlogram* functions associated to Moran's I tests (R package *spatial*; Moran, 1950; Table S4.7), as well as any obvious problem in the overdispersion ratio (0.8–1.4; Table 2) on the best models. Relative variance explained by each fixed effect (*pseudo R*²) was calculated from generalized linear models, because it is not covered by recent computing methods for GLMMs using a negative binomial distribution. Models were validated by visual examination of residuals plots. All analyses were performed using a significant threshold of 5% in R statistical software v.3.3.1.

**Table 2:** Description of the dataset for each response variable from the 64 sites, the number of bat passes, occurrences (% of sites for which species were recorded), the best models from the multimodel inference procedure, and the response variable distribution selected (NB: negative binomial; θ: overdispersion ratio). Full models are shown in Table S4.3

Response variable	No. of bat passes (mean per night)	Occurrences					Best model	Distribution
		Total	OT	CT	CTH	T		
*Pipistrellus kuhlii*	68 (1.0)	36	67	54	33	10	System + dist. to roads + (1|date)	NB (θ = 1.2)
*P. nathusii*	79 (1.3)	34	67	46	33	10	(1|date)	NB (θ = 1.3)
*P. pipistrellus*	1,125 (17.5)	67	100	92	61	38	System + dist. to roads + (1|date)	NB (θ = 1.1)
*Pipistrellus* spp.	1272 (6.6)	69	78	64	43	19	System + dist. to roads + (1|date) + (1|species)	NB (θ = 1.0)
*Nyctalus* spp.	48 (0.8)	11	8	31	0	10	System + (1|date)	Binomial (θ = 0.8)
Richness	–	–	–	–	–	–	System + (1|date)	NB (θ = 1.4)

OT, organic tillage fields; CT, conservation tillage fields; CTH, conservation tillage fields using more herbicide; T, tillage fields.

## 3 RESULTS

### 3.1 Bat monitoring

We recorded 1,328 bat passes from five species (*P. pipistrellus*,* P. kuhlii*,* P. nathusii*,* Nyctalus noctula*, and *Nyctalus leisleri*) and two genera (*Myotis* spp. and *Plecotus* spp.; i.e., an overall species richness of seven) in the 64 study sites, where the most abundant species was *P. pipistrellus* representing 85% of the total activity. The two species groups *Plecotus* spp. and *Myotis* spp. were the least abundant (respectively, two and six bat passes), detected in 8% and 3% of sites, respectively (Table S4.8). The two species *N. noctula* and *N. leisleri* were grouped for analyses in *Nyctalus* spp., thanks to their similar ecological niche and their, respectively, low activity. Only three species and one genus (*Nyctalus* spp.) were therefore present in a sufficient number of sites for analyses (Table 2).

### 3.2 Selected candidate models

The system type variable was selected in all candidate models with a delta AICc <2 for *P. kuhlii*,* P. pipistrellus*,* Pipistrellus* spp., and richness, only twice for four candidate models for *Nyctalus* spp., and none for *P. nathusii* (Table S4.6). For *P. nathusii*, we retained the system type variable from the best model (i.e., null model) in analyses to obtain estimated parameters and predicted activity for systems.

### 3.3 Effect of farming systems

In comparison with OT, CTH and T systems exhibit a significantly lower activity of *P. kuhlii* (Table 3; Figure 2a), *P. pipistrellus* (Table 3; Figure 2b), *Pipistrellus* spp. (Table 3; Figure 2d), and richness (Table 3). For all species, we did not find activity differences between OT and CT systems (Table 3).

**Table 3:** Estimates and standard errors for farming systems comparisons when OT (A), CT (B), and T (C) are used as the intercept, and distance environmental covariates from the averaging of candidate models having a delta AICc <2 (***p < .001, **p < .01, *p < .05, *p* < .1)

	*Pipistrellus kuhlii*	*P. nathusii*	*P. pipistrellus*	*Pipistrellus* spp.	*Nyctalus* spp.	Richness
Farming systems						
(A) CT versus OT	−0.12 (0.67)	−0.27 (0.85)	−0.67 (0.71)	−0.36 (0.46)	1.44 (1.24)	−0.08 (0.27)
(A) CTH versus OT	−1.68 (0.69)*	−0.36 (0.80)	−1.54 (0.63)*	−1.20 (0.41)**	/	−0.70 (0.28)*
(A) T versus OT	−1.59 (0.68)*	−1.10 (0.80)	−2.42 (0.64)***	−2.10 (0.43)***	0.01 (1.31)	−1.30 (0.33)***
(B) CTH versus CT	−1.57 (0.66)*	−0.09 (0.79)	−0.87 (0.65)	−0.84 (0.44)	/	−0.62 (0.28)*
(B) T versus CT	−1.48 (0.68)*	−0.78 (0.79)	−1.75 (0.67)**	−1.70 (0.45)***	−1.44 (0.96)	−1.22 (0.33)***
(C) CTH versus T	−0.09 (0.69)	0.69 (0.72)	0.88 (0.58)	0.85 (0.41)*	/	0.60 (0.35)
Covariates						
Dist. to roads	−0.50 (0.26)	−0.33 (0.25)	−0.56 (0.23)*	−0.54 (0.15)***	/	−0.15 (0.11)
Dist. to hedgerows	−0.39 (0.28)	0.17 (0.27)	/	−0.18 (0.21)	/	−0.11 (0.13)
Dist. to boundaries	/	−0.24 (0.24)	/	/	/	/
Dist. to forests	/	/	/	/	−0.53 (0.49)	/
Dist. to wetlands	/	/	/	/	/	−0.10 (0.14)

OT, organic tillage fields; CT, conservation tillage fields; CTH, conservation tillage fields using more herbicide; T, tillage fields.

Within conventional systems, CTH and T systems exhibit a significantly lower activity of *P. kuhlii* (Table 3; Figure 2a) and richness (Table 3) than CT. T systems showed a significantly lower activity of *P. pipistrellus* (Table 3; Figure 2b) and *Pipistrellus* spp. (Table 3; Figure 2d) than CT. Similarly, compared to CTH, only the activity of *Pipistrellus* spp. was lower in T systems (Table 3; Figure 2d). No differences between farming systems were found for *P. nathusii* and *Nyctalus* spp. (Table 3; Figure 2c), and only the distance to roads among covariates was significant for *P. pipistrellus* and *Pipistrellus* spp. (Table 3).

Finally, farming systems always explained the most relative part of the variance compared to other covariates of full models (Table S4.9).

## 4 DISCUSSION

To our knowledge, this study is the first to assess the effect of accurate farming practices on bats, comparing the effect of organic and a gradient of conventional systems in tillage and herbicide intensity. We tested differences in farming practices the same year, on one crop type and in contiguous fields, in a homogeneous intensive landscape, allowing the study of the basic pathways through which agriculture affects the bat community and thus complementing previous farm‐level approaches (Wickramasinghe et al., 2003). Our results highlight that the organic tillage system (OT) always had a significantly greater positive effect on bats than the tillage system (T, conventional farming). The differences (1) in the farming practices and (2) in bat activity between these two systems suggested that pesticides in T had an important negative effect on bats. The conservation tillage system using more herbicide (CTH, conventional farming) also had negative effects compared to OT, suggesting that the possible positive effects of conservation tillage present in CTH (compared to T) did not mitigate the negative effects of herbicides. These differences between the organic and the two conventional systems are in accordance with previous results (Fuller et al., 2005; Wickramasinghe et al., 2003), even if the practice features were not explicitly taken into account in these previous studies. However, we did not detect differences between the OT and the conservation tillage systems (CT, conventional farming). This major result suggests that it may be possible to approach the positive effects of organic farming in conventional farming thanks to the reduction of herbicides and the use of conservation tillage. In addition, it should be noted that our results indirectly revealed the respective negative effects of tillage and herbicide intensification in the four systems. Indeed, in conventional systems, tillage (i.e., inversion of soil to 30‐cm depth, such as in T) appears to be less attractive for bats than conservation tillage (i.e., superficial tillage only such as in CT and CTH). Similarly, a conservation tillage system as well as a tillage system appeared to be less attractive when more herbicide was applied, such as suggested by the comparison of bat activity OT versus T and CT versus CTH.

### 4.1 Mechanism hypotheses, limitations, and perspectives

We hypothesized that resource limitation drives the foraging selection of generalist bat species within studied fields and could explain the results depending on the diet, composed of arthropods for European species (Vaughan, 1997). Because the fields are all wheat, other influences such as structural heterogeneity did not seem to be important. For a given farming system, diversity of taxa (Arachnida, Coleoptera, and Diptera) are less abundant in tillage than in conservation tillage (Holland & Reynolds, 2003; Rodríguez et al., 2006). In addition, herbicides used in higher quantities negatively and indirectly affect the structure and diversity of the arthropod community through food resource, host plant availability, and habitat modifications (Bitzer, Buckelew, & Pedigo, 2002; Geiger et al., 2010; Taylor et al., 2006; Wardle, Nicholson, Bonner, & Yeates, 1999). Herbicides can also cause negative direct‐impacts on Arachnida and Coleoptera behavior and survival (Evans et al., 2010). According to the differences in diet composition among bat species, aerial hawkers such as *Nyctalus* spp and *Pipistrellus* spp forage proportionally more on flying insects (i.e., moths and Diptera) than gleaner species such as *Myotis* spp and *Plecotus* spp, more often specialized in ground beetle or spiders (Vaughan, 1997). Future studies should attempt to simultaneously measure variation among arthropod community availability and bat activity linked to tillage and herbicide intensity.

Our study was conducted on a small scale, both temporally and spatially, requiring further studies in different landscape contexts and other countries. However, these limitations appearing as a weakness also provides serious advantages. First, bat taxa are known for substantial internight, seasonal, and yearly variations in abundances, thus a sampling design within a short period allows to minimize this temporal source variance. In addition, sampled nights were performed during the lactating period, a season with high energetic constraints. Secondly, the small spatial scale of this study allowed an avoidance of variance source connected to the landscape. Our sampling design allowed us to study in detail the choice of plots by individuals, thanks to continuity or proximity between plots. Recorded individuals had the ability to choose a plot of a given system rather than another, this demonstrates a plot selection that is not influenced by landscape characteristics, distance to roost or landscape connectivity.

### 4.2 Application perspectives

The diversification of practices in organic systems allows the reduction in the yield gap with conventional farming (Ponisio et al., 2015), and organic farming can become more comparable economically to conventional farming (Crowder & Reganold, 2015). Despite this, the switch of conventional to organic farming is often limited by a lack of knowledge in production methods, unsuitable technical infrastructure and marketing, low buying power and government policies (Reganold et al., 2011). Although organic systems and their more biodiversity‐friendly practices are developing, the surface they could cover within a few years may not be sufficient to significantly reduce the erosion of biodiversity in agricultural systems (even if organic systems have increased by 150% over the decade 2004–2014, they only cover 4.9% of total arable crops in Europe; Eurostat, 2015; FiBL, 2014). Despite this, our study demonstrates that conventional systems can still benefit biodiversity; thus, it is important to widely implement alternative practices in favor of biodiversity in conventional farming for the 95.1% of remaining arable crops. Among the several possibilities of changes in practice, the characteristics of the studied CT system appear promising to approach the benefits of the organic system for bats. This system is, in addition, equally productive to the other studied systems in conventional farming, between 9 and 11 t/ha in recent years. Indeed, alternative practices in conventional farming such as the reduction of herbicide use is not antagonistic to production (Petit et al., 2015) and may even be reduced by 37% while preserving arable crop productivity and profitability (Lechenet, Dessaint, Py, Makowski, & Munier‐jolain, 2017), which is consistent with the studied CT system using one less herbicide among the three used in CTH systems. Thus, even if organic farming appears as the best method for bat conservation in agricultural systems, it could be a great step in the actual context of the very low level of organic system representation to undertake a transition in conventional farming from intensive to more biodiversity‐friendly practices such as shown in this study. These findings have important implications for biodiversity conservation in the agricultural landscape on a larger scale, as studied practice changes were performed on widespread conventional systems.

## CONFLICT OF INTEREST

None declared.

## AUTHOR CONTRIBUTIONS

KB and FC conceived the ideas, KB designed the methodology and collected the data; KB and CK analyzed the data; all authors led the writing of the manuscript. All authors critically contributed to the drafts and gave their final approval for publication.

## DATA ACCESSIBILITY

Data are available on Dryad Digital Repository (https://doi.org/10.5061/dryad.s53ns).
