# Supplementary material for: Tillage and herbicide reduction mitigate the gap between conventional and organic farming effects on foraging activity of insectivorous bats
Source: Ecol Evol. 2017 Dec 30;8(3):1496–506. doi: 10.1002/ece3.3688 (PMC5792571; doi:10.1002/ece3.3688)
Supplement: Supplementary file 1 [file ECE3-8-1496-s001.doc]

*Tillage and herbicide reduction mitigate the gap between conventional and organic farming effects on insectivorous bats*

*Kévin Barré, Isabelle Le Viol, Romain Julliard, François Chiron and Christian Kerbiriou*

**Supplementary information**

**Appendix S1. Agricultural context of study**

Table S1.1. Mean agricultural yields in 2015 of the main crops in France and Ile-de-France region compared to European Union.

| Crop types | Agricultural yield (100 kg/ha) | | |
| --- | --- | --- | --- |
|  | Europe | France | Ile-de-France region |
| barley | 61.97 | 63.30 | 67.05 |
| corn | 72.23 | 90.5 | 100.21 |
| pea | 28.26 | 45.12 | 45.27 |
| rape | 29.81 | 29.30 | 29.49 |
| sugar beet | 679.45 | 826.89 | 809.28 |
| wheat | 54.75 | 72.64 | 81.33 |

**References**

Eurostat, 2015. European statistics explained. Available at: <http://appsso.eurostat.ec.europa.eu/nui/submitViewTableAction.do>

Agreste, 2015. Ministère de l'Agriculture, de l’Alimentation, de la Pêche, de la Ruralité, et de l'Aménagement du Territoire. Available at: <https://stats.agriculture.gouv.fr/disar/faces/report/welcomeReport.jsp>

Table S1.2. Proportion of the main land cover in Ile-de-France region and study site compared to France.

| Land use | France | Ile-de-France region | | Study site |
| --- | --- | --- | --- | --- |
| agricultural areas (mainly, arable land) | 53% | 59% | 57% | |
| forest and semi natural areas | 35% | 22% | 16% | |
| artificial surfaces | 9% | 18% | 25% | |
| wetlands and water bodies | 3% | 1% | 2% | |

Table S1.3. Proportion of the Utilized Agricultural Land (UAL) and trends in organic farming in France and Ile-de-France region compared to Europe.

| Organic farming type | Europe | |  | France | |  | Ile-de-France region | |  | Study site |
| --- | --- | --- | --- | --- | --- | --- | --- | --- | --- | --- |
|  | % UAL | 2011-2015 trend (%) |  | % UAL | 2011-2015 trend (%) |  | % UAL | 2011-2015 trend (%) |  | % UAL |
| Arable crops | 4.9 | / |  | 2.5 | + 52.7 |  | 1.4 | / |  | 4.1 |
| Total | 2.4 | + 21.1 |  | 4.9 | + 58.6 |  | 2.0 | + 46.5 |  | 4.1 |

**References**

Agence Bio, 2016. Agence Française pour le Développement et la Promotion de l’Agriculture Biologique. Available at: <http://www.agencebio.org/sites/default/files/upload/documents/4_Chiffres/BrochureCC/CC2016_TERRITOIRES.pdf>

Agence Bio, 2015. Agence Française pour le Développement et la Promotion de l’Agriculture Biologique. Available at: <http://www.agencebio.org/sites/default/files/upload/documents/4_Chiffres/BrochureCC/Regions/CC_fiche_IDF.pdf>

FiBL-IFOAM, 2014. Organic Farming Statistics. Research Institute of Organic Agriculture FiBL, Available at: <https://shop.fibl.org/fileadmin/documents/shop/1698-organic-world-2016.pdf>

Eurostat, 2013. European statistics explained. Available at: <http://ec.europa.eu/eurostat/statistics-explained/index.php/File:Utilised_agricultural_area,_by_land_use,_2010_(1_000_hectares)_AgriPB13.png>

Eurostat, 2015. European statistics explained. Available at: <http://ec.europa.eu/eurostat/statistics-explained/index.php/Organic_farming_statistics>

Table S1.4. Proportion of the three types of soil management in arable and wheat crops for France, Ile-de-France and study site.

| Type of soil management | France | |  | Ile-de-France region | |  | Study site | |
| --- | --- | --- | --- | --- | --- | --- | --- | --- |
|  | Arable land | Wheat |  | Arable land | Wheat |  | Arable land | Wheat |
| Tillage | 70.1 | 56.0 |  | 78.6 | / |  | 85.7 | / |
| Conservation tillage | 28.4 | 39.6 |  | 21.4 | / |  | 14.3 | / |
| Direct-seeding | 1.4 | / |  | / | / |  | 0 | 0 |

Data is missing in Agreste (2011) dataset for France and Ile-de-France region and not available for the study site as although types of soil management were known on all fields of the study area, crop rotations were not.

Tillage is a classic ploughing method (inversion of soil to a depth of a minimum of 30 cm), conservation tillage is a no-ploughing method comprising of a superficial tillage without destructuring the soil (often harrowing and smoothing), and direct-seeding is a complete no-tillage method (whether deep or superficial).

**Reference**

Agreste, 2011. Ministère de l'Agriculture, de l’Alimentation, de la Pêche, de la Ruralité, et de l'Aménagement du Territoire. Available at: <https://stats.agriculture.gouv.fr/disar/faces/report/welcomeReport.jsp>
