# Supplementary material for: Tillage and herbicide reduction mitigate the gap between conventional and organic farming effects on foraging activity of insectivorous bats
Source: Ecol Evol. 2017 Dec 30;8(3):1496–506. doi: 10.1002/ece3.3688 (PMC5792571; doi:10.1002/ece3.3688)
Supplement: Supplementary file 2 [file ECE3-8-1496-s002.doc]

*Tillage and herbicide reduction mitigate the gap between conventional and organic farming effects on insectivorous bats*

*Kévin Barré, Isabelle Le Viol, Romain Julliard, François Chiron and Christian Kerbiriou*

**Supplementary information**

**Appendix S2. Landscape composition around recording sites**


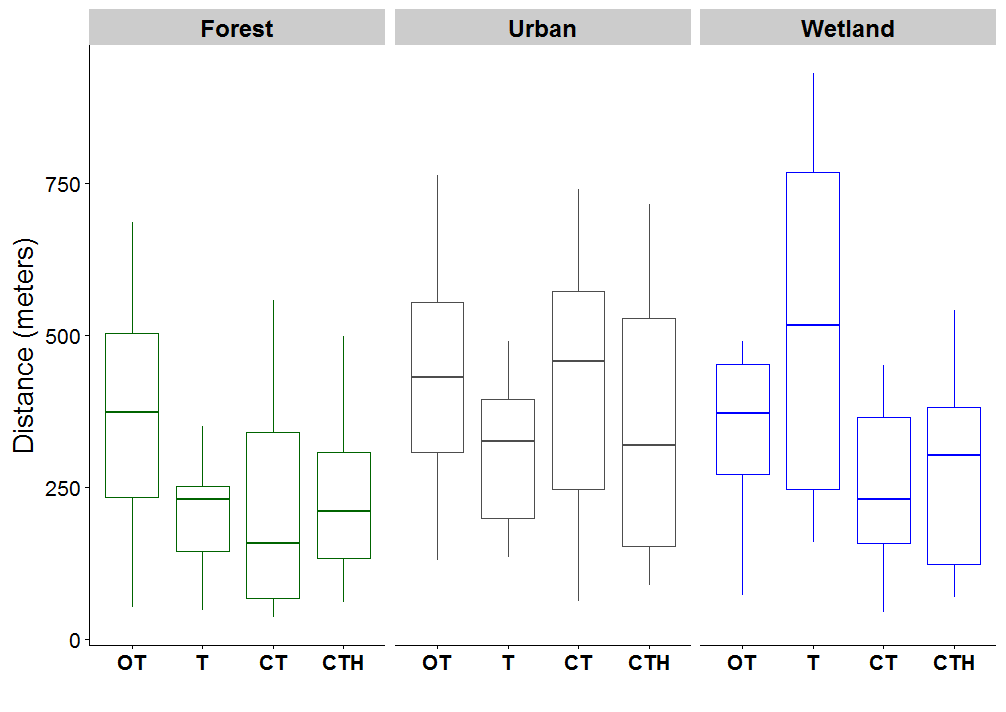


Fig. S2.1. Distance of sampling sites according to the farming system (OT: organic tillage; CT: conservation tillage fields; CTH: conservation tillage fields using more herbicide; T: tillage) to the nearest landscape elements (Forest, Urban and Wetland).
