# Supplementary material for: Tillage and herbicide reduction mitigate the gap between conventional and organic farming effects on foraging activity of insectivorous bats
Source: Ecol Evol. 2017 Dec 30;8(3):1496–506. doi: 10.1002/ece3.3688 (PMC5792571; doi:10.1002/ece3.3688)
Supplement: Supplementary file 3 [file ECE3-8-1496-s003.doc]

*Tillage and herbicide reduction mitigate the gap between conventional and organic farming effects on insectivorous bats*

*Kévin Barré, Isabelle Le Viol, Romain Julliard, François Chiron and Christian Kerbiriou*

**Supplementary information**

**Appendix S3. Characteristics of farming systems**

Table S3.1. Quantities of active substances of herbicides used in three conventional systems according to month of application. Note that for a given system and month, all herbicides are used in combination (e.g. Toiseau + Spow in September for CTH system).

| **Month of application** | **Trade names** | **Active substances** | **Quantity (L/Ha)** | | |
| --- | --- | --- | --- | --- | --- |
|  |  |  | **CT** | **CTH** | **T** |
| ***September*** |  |  |  |  |  |
|  | Toiseau | Diflufenicanil | / | 0.3 | / |
|  | Fosburi | Diflufenicanil + Flufenacet | / | / | / |
|  | Aubaine | Isoxaben + Chlorotoluron | / | / | / |
|  | Defi | Metsulfuron-methyl | / | / | / |
|  | Spow | Prosulfocarb | / | 2.0 | / |
| ***October*** |  |  |  |  |  |
|  | Toiseau | Diflufenicanil | / | / | / |
|  | Fosburi | Diflufenicanil + Flufenacet | / | / | 0.4 |
|  | Aubaine | Isoxaben + Chlorotoluron | 1.5 | 1.5 | 1.5 |
|  | Defi | Metsulfuron-methyl | 2.5 | 2.5 | 2.5 |
|  | Spow | Prosulfocarb | / | / | / |
| ***March*** |  |  |  |  |  |
|  | Toiseau | Diflufenicanil | / | / | / |
|  | Fosburi | Diflufenicanil + Flufenacet | 0.4 | 0.4 | 0.4 |
|  | Aubaine | Isoxaben + Chlorotoluron | / | / | / |
|  | Defi | Metsulfuron-methyl | 2.5 | 2.5 | 2.5 |
|  | Spow | Prosulfocarb | / | / | / |
| ***Molecular formula***  *Chlorotoluron: C10H13ClN2O; Diflufenicanil: C19H11F5N2O2; Flufenacet: C14H13F4N3O2S; Isoxaben: C18H24N2O4; Metsulfuron-methyl: C14H15N5O6S; Prosulfocarb: C14H21NOS* | | | | | |
| Aubaine will be prohibited from 2018. | | |  |  |  |

Table S3.2. Quantities of active substances of fungicides used in three conventional systems according to month of application. Note that for a given month, fungicide was used alone or in combination (i.e. Sunorg pro + Prochlosun + Fongil; Librax + Cinch pro + Tazer; Piano; Xtra + Cherokee; Librax + Comet; Prosaro).

| **Month of application** | **Trade names** | **Active substances** | **Quantity (L/Ha)** | | | |  | | | | | | | |
| --- | --- | --- | --- | --- | --- | --- | --- | --- | --- | --- | --- | --- | --- | --- |
|  |  |  | **CT** | **CTH** | **T** | |  | | | | | | | |
| ***April*** |  |  |  |  |  | |  | | | | | | | |
|  | Fongil | Chlorothalonil | 0.5 | / | 0.5 | |  | | | | | | | |
|  | Cherokee | Chlorothalonil + Cyproconazole + Propiconazole | 1.0 | 1.0 | 1.0 | |  | | | | | | | |
|  | Xtra | Cyproconazol + Azoxystrobin | 0.3 | 0.3 | 0.3 | |  | | | | | | | |
|  | Sunorg pro | Metconazole | 0.5 | / | 0.5 | |  | | | | | | | |
|  | Prochlosun | Prochloraz | 0.5 | / | 0.5 | |  | | | | | | | |
| ***May*** |  |  |  |  |  | |  | | | | | | | |
|  | Tazer | Azoxystrobin | 0.2 | / | 0.2 | |  | | | | | | | |
|  | Cinch pro | Metconazole | 0.2 | / | 0.2 | |  | | | | | | | |
|  | Librax | Metconazole + Fluxapyroxad | 1.0 | 1.0 | 1.0 | |  | | | | | | | |
|  | Comet | Pyraclostrobin | 0.3 | 0.3 | 0.3 | |  | | | | | | | |
| ***June*** |  |  |  |  |  | |  | | | | | | | |
|  | Piano | Prothioconazole + Tebuconazole | 0.4 | / | 0.4 | |  | | | | | | | |
|  | Prosaro | Prothioconazole + Tebuconazole | 0.7 | 0.7 | 0.7 | |  | | | | | | | |
| ***Molecular formula***  *Azoxystrobin: C22H17N3O5; Chlorothalonil: C8Cl4N2; Cyproconazol: C15H18ClN3O; Fluxapyroxad: C18H12F5N3O; Metconazole: C17H22ClN3O; Prochloraz: C15H16Cl3N3O2; Propiconazole: C15H17Cl2N3O2; Prothioconazole: C14H15Cl2N3OS; Pyraclostrobin: C19H18ClN3O4; Tebuconazole: C16H22ClN3O* | | | | | |  | |  | | | |  |  |  |
|  | | | | | | |  | |  |  |  | | | |
|  | | |  |  |  | |  | | | | | | | |
|  | | | | | | |  | | | | | | | |

Table S3.3. Previous crop of wheat fields studied, number of fields and number of sampling sites (in parentheses) according to farming systems (OT: organic tillage fields; CT: conservation tillage fields; CTH: conservation tillage fields using more herbicide; T: tillage fields). In all systems wheat crops are implemented every two years followed by field bean or lucerne in OT system; maize, field bean, barley or rape for CT system; field bean or rape for CTH system; maize, field bean or barley for T system. However, the lucerne in OT system is implemented every three years. Organic fields are established for more than ten years, conventional and conservation tillage is performed on different fields each year according to the last crop and weather conditions in August to October. The last crops were field bean and lucerne for OT system, field bean and rape for CT system, rape and field bean for CTH system, maize and field bean for T system.

| **Farming systems** | **Last crops of wheat fields in farming systems** | | | | |
| --- | --- | --- | --- | --- | --- |
|  | Field bean | Rape | Barley | Maize | Lucerne |
| OT *no. of fields (sites)* | x 1 (8) |  |  |  | x 1 (4) |
| CT  *no. of fields (sites)* | x 3 (8) | x 2 (5) | x |  |  |
| CTH *no. of fields (sites)* | x 1 (1) | x 4 (17) |  |  |  |
| T *no. of fields (sites)* | x 2 (4) |  | x | x 5 (17) |  |
