# Supplementary material for: Tillage and herbicide reduction mitigate the gap between conventional and organic farming effects on foraging activity of insectivorous bats
Source: Ecol Evol. 2017 Dec 30;8(3):1496–506. doi: 10.1002/ece3.3688 (PMC5792571; doi:10.1002/ece3.3688)
Supplement: Supplementary file 4 [file ECE3-8-1496-s004.doc]

*Tillage and herbicide reduction mitigate the gap between conventional and organic farming effects on insectivorous bats*

*Kévin Barré, Isabelle Le Viol, Romain Julliard, François Chiron and Christian Kerbiriou*

**Supplementary information**

**Appendix S4. Additional results concerning bat monitoring, acoustic data validation and statistical analyses**

*Table S4.1. Number of sampled sites per night between 16 June and 23 June 2016 (OT: organic tillage fields; CT: conservation tillage fields; CTH: conservation tillage fields using more herbicide; T: tillage fields).*

|  | **Number of sampled sites/night** | | | | | | | | |
| --- | --- | --- | --- | --- | --- | --- | --- | --- | --- |
| **Farming systems** | 06/16 | 06/17 | 06/18 | 06/19 | 06/20 | 06/21 | 06/22 | 06/23 | Total |
| OT | 3 | 2 | 2 | 2 | 1 | 1 | 1 | 0 | 12 |
| CT | 2 | 2 | 2 | 2 | 2 | 1 | 1 | 1 | 13 |
| CTH | 3 | 1 | 4 | 2 | 3 | 2 | 2 | 1 | 18 |
| T | 1 | 2 | 3 | 4 | 3 | 2 | 2 | 4 | 21 |
| Total | 9 | 7 | 11 | 10 | 9 | 6 | 6 | 6 | 64 |

Table S4.2. Summary of species assigned to bat passes in TADARIDA software among error probability, and final no. of bat passes used in models resulting from manual validations.

| **TADARIDA results** | | |  | **Manual validations** | | |  | **Real no. of bat passes** | |
| --- | --- | --- | --- | --- | --- | --- | --- | --- | --- |
| Species | Probability classes | No.bat passes |  | checked % | % of errors | correction |  |  |  |
| *Eptesicus serotinus* | 0.5 - 0.6 | 1 |  | 100 | 100 | Nyclei (1) |  | 0 | 0 |
|  | 0.6 - 0.7 | 7 |  | 100 | 100 | Nyclei (7) |  | 0 |  |
|  | 0.7 - 0.8 | 6 |  | 100 | 100 | Nyclei (6) |  | 0 |  |
| *Hypsugo savii* | 0.6 - 0.7 | 1 |  | 100 | 100 | Pipkuh (1) |  | 0 | 0 |
| *Myotis capaccinii* | 0.2 - 0.3 | 1 |  | 100 | 100 | Plecsp (1) |  | 0 | 0 |
| *Myotis daubentonii* | 0.5 - 0.6 | 3 |  | 100 | - | Myosp (3) |  | 0 | 0 |
|  | 0.6 - 0.7 | 1 |  | 100 | - | Myosp (1) |  | 0 |  |
| *Myotis nattereri* | 0.6 - 0.7 | 1 |  | 100 | - | Myosp (1) |  | 0 | 0 |
|  | 0.9 - 1 | 1 |  | 100 | - | Myosp (1) |  | 0 |  |
| *Myotis ssp* | 0.5 - 0.6 | - |  | - | - | - |  | 3 | 6 |
|  | 0.6 - 0.7 | - |  | - | - | - |  | 2 |  |
|  | 0.9 - 1 | - |  | - | - | - |  | 1 |  |
| *Nyctalus leisleri* | 0.5 - 0.6 | - |  | - | - | - |  | 1 | 16 |
|  | 0.6 - 0.7 | - |  | - | - | - |  | 7 |  |
|  | 0.7 - 0.8 | 2 |  | 100 | 0 | - |  | 8 |  |
| *Nyctalus noctula* | 0.5 - 0.6 | 7 |  | 100 | 0 | - |  | 7 | 32 |
|  | 0.6 - 0.7 | 5 |  | 100 | 0 | - |  | 5 |  |
|  | 0.7 - 0.8 | 17 |  | 100 | 0 | - |  | 17 |  |
|  | 0.8 - 0.9 | 3 |  | 100 | 0 | - |  | 3 |  |
| *Pipistrellus kuhlii* | 0.2 - 0.3 | 1 |  | 100 | 0 | - |  | 1 | 68 |
|  | 0.3 - 0.4 | - |  | - | - | - |  | 1 |  |
|  | 0.5 - 0.6 | 13 |  | 100 | 0 | - |  | 17 |  |
|  | 0.6 - 0.7 | 30 |  | 100 | 0 | - |  | 39 |  |
|  | 0.7 - 0.8 | 5 |  | 100 | 0 | - |  | 10 |  |
| *Pipistellus nathusii* | 0.3 - 0.4 | 1 |  | 100 | 100 | Pipkuh (1) |  | 0 | 79 |
|  | 0.4 - 0.5 | 1 |  | 100 | 0 | - |  | 1 |  |
|  | 0.5 - 0.6 | 17 |  | 100 | 47.1 | Pippip (4); Pipkuh (4) |  | 9 |  |
|  | 0.6 - 0.7 | 44 |  | 100 | 54.5 | Pippip (13); Pipkuh (5) |  | 20 |  |
|  | 0.7 - 0.8 | 57 |  | 100 | 33.3 | Pippip (14); Pipkuh (8) |  | 38 |  |
|  | 0.8 - 0.9 | 11 |  | 100 | 0 | - |  | 11 |  |
| *Pipistrellus pipistrellus* | 0.3 - 0.4 | 2 |  | 100 | 0 | - |  | 2 | 1125 |
|  | 0.4 - 0.5 | 3 |  | 100 | 0 | - |  | 3 |  |
|  | 0.5 - 0.6 | 62 |  | 20 | 0 | - |  | 66 |  |
|  | 0.6 - 0.7 | 178 |  | 20 | 0 | - |  | 192 |  |
|  | 0.7 - 0.8 | 260 |  | 20 | 0 | - |  | 273 |  |
|  | 0.8 - 0.9 | 411 |  | 20 | 0 | - |  | 411 |  |
|  | 0.9 - 1 | 178 |  | 20 | 0 | - |  | 178 |  |
| *Plecotus austriacus* | 0.6 - 0.7 | 1 |  | 100 | - | Plecsp (1) |  | 0 | 0 |
| *Plecotus ssp* | 0.2 - 0.3 | - |  | - | - | - |  | 1 | 2 |
|  | 0.6 - 0.7 | - |  | - | - | - |  | 1 |  |

(Nyclei: *Nyctalus leisleri*; Pipkuh: *Pipistrellus kuhlii*; Pippip: *Pipistrellus pipistrellus*; Plecsp: *Plecotus ssp*; Myosp: *Myotis ssp*)

Using BatSound© software version 4.03, we checked by screening all calls of species assigned in TADARIDA software, except for *Pipistrellus pipistrellus* where all calls between 0 to 0.4 confidence indices and randomly 20% of each other 0.1 indices classes were checked. When nothing is written in “manual validations” and “TADARIDA results”, bat passes come from errors in other species (i.e. *Nyctalus leisleri* and *Pipistrellus kuhlii*) or impossibility of determination at species level (i.e. *Myotis ssp* and *Plecotus ssp*).

Table S4.3. Full composition of each species, genera and richness models. In bold are indicate correlated variables which were not included together during the multi-model inference using the dredge function in R.

| Dependant variables | Full models |
| --- | --- |
| *Pipistrellus kuhlii* | system + **hedgerows** + roads + **wetlands** + (1|date) |
| *Pipistrellus nathusii* | system + **boundaries** + **roads** + hedgerows + (1|date) |
| *Pipistrellus pipistrellus* | system + **roads** + **boundaries** + wetlands + (1|date) |
| *Pipistrellus ssp.* | system + **wetlands** + roads + **hedgerows** + (1|date) + (1|species) |
| *Nyctalus ssp.* | system + **wetlands** + forests + **hedgerows** + (1|date) |
| Richness | system + **wetlands** + roads + **hedgerows** + (1|date) |

Table S4.4. Results of Kruskal-Wallis tests between land-use and farming system variables. Note that significant relations in bold did not cause problems in VIF analysis performed (< 2 for each covariable) of full models.

| Kruskal-Wallis test | | |
| --- | --- | --- |
|  | ks | p-value |
| Distance to roads | 1.754 | 0.625 |
| Distance to boundaries | 1.722 | 0.632 |
| Distance to hedgerows | **10.050** | **0.018** |
| Distance to wetlands | **14.287** | **0.003** |
| Distance to forests | 6.454 | 0.091 |
| Distance to urban areas | 4.983 | 0.173 |

Table S4.5. Correlation coefficients of covariables included in the analysis.

|  | Wetlands | Hedgerows | Urban areas | Boundaries | Roads |
| --- | --- | --- | --- | --- | --- |
| Forests | 0.001 | 0.014 | 0.319 | -0.126 | -0.184 |
| Wetlands |  | **0.763** | 0.208 | 0.122 | 0.104 |
| Hedgerows |  |  | 0.133 | 0.095 | 0.114 |
| Urban areas |  |  |  | 0.304 | 0.287 |
| Boundaries |  |  |  |  | **0.945** |


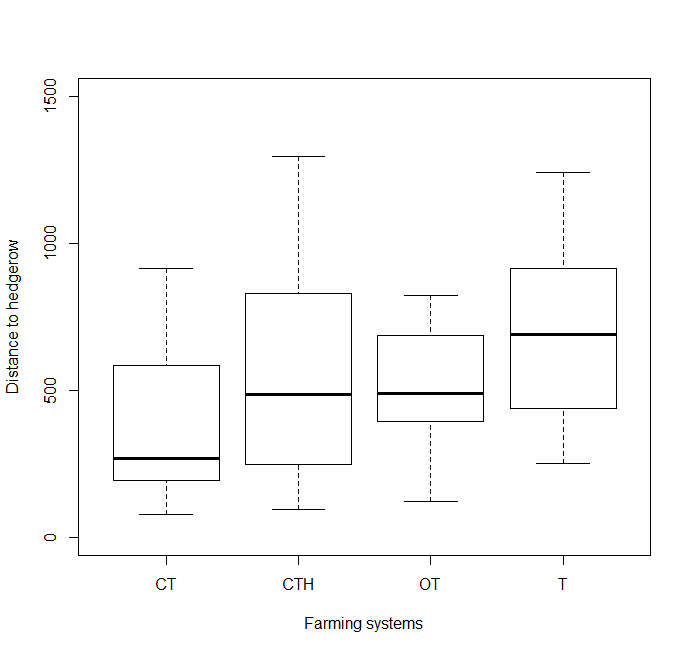


Fig. S4.1. Distance of sampling sites to hedgerows according to the farming system (OT: organic tillage; CT: conservation tillage fields; CTH: conservation tillage fields using more herbicide; T: tillage).

Table S4.6: results of multi-model inference ranked by delta AICc < 2, for which correlated covariables (distance to hedgerows/wetlands and distance to roads/boundaries) were not included simultaneously.

| Model number | Intercept | System | Forest | Boundaries | Hedgerow | Wetland | Road | DF | AICc | delta AICc | weight |
| --- | --- | --- | --- | --- | --- | --- | --- | --- | --- | --- | --- |
| ***Nyctalus ssp.*** |  |  |  |  |  |  |  |  |  |  |  |
| 2 | -2.40 | + |  |  |  |  |  | 5 | 47.20 | 0.00 | 0.25 |
| 1 | -2.10 |  |  |  |  |  |  | 2 | 48.40 | 1.21 | 0.14 |
| 4 | -2.16 | + | -0.49 |  |  |  |  | 6 | 48.50 | 1.29 | 0.13 |
| 3 | -2.22 |  | -0.59 |  |  |  |  | 3 | 49.00 | 1.85 | 0.10 |
| ***Pipistrellus kuhlii*** | |  |  |  |  |  |  |  |  |  |  |
| 6 | 0.71 | + |  |  |  |  | -0.51 | 7 | 171.30 | 0.00 | 0.29 |
| 8 | 0.69 | + |  |  | -0.37 |  | -0.47 | 8 | 172.20 | 0.87 | 0.19 |
| 4 | 0.54 | + |  |  | -0.43 |  |  | 7 | 173.10 | 1.77 | 0.12 |
| 2 | 0.56 | + |  |  |  |  |  | 6 | 173.10 | 1.79 | 0.12 |
| ***Pipistrellus nathusii*** | |  |  |  |  |  |  |  |  |  |  |
| 1 | 0.22 |  |  |  |  |  |  | 3 | 184.60 | 0.00 | 0.28 |
| 9 | 0.17 |  |  |  |  |  | -0.33 | 4 | 185.30 | 0.69 | 0.20 |
| 3 | 0.19 |  |  | -0.24 |  |  |  | 4 | 185.90 | 1.36 | 0.14 |
| 5 | 0.21 |  |  |  | 0.17 |  |  | 4 | 186.50 | 1.89 | 0.11 |
| ***Pipistrellus pipistrellus*** | |  |  |  |  |  |  |  |  |  |  |
| 6 | 3.59 | + |  |  |  |  | -0.57 | 7 | 445.60 | 0.00 | 0.54 |
| ***Pipistrellus ssp.*** |  |  |  |  |  |  |  |  |  |  |  |
| 6 | 1.62 | + |  |  |  |  | -0.55 | 8 | 785.20 | 0.00 | 0.55 |
| 8 | 1.64 | + |  |  | -0.18 |  | -0.52 | 9 | 786.70 | 1.47 | 0.26 |
| **Richness** |  |  |  |  |  |  |  |  |  |  |  |
| 2 | 0.98 | + |  |  |  |  |  | 6 | 211.60 | 0.00 | 0.34 |
| 6 | 1.00 | + |  |  |  |  | -0.15 | 7 | 212.30 | 0.70 | 0.24 |
| 4 | 0.96 | + |  |  | -0.11 |  |  | 7 | 213.30 | 1.73 | 0.14 |
| 10 | 0.96 | + |  |  |  | -0.10 |  | 7 | 213.60 | 1.99 | 0.13 |

Table S4.7. Results of checking for potential spatial autocorrelation, using Moran’s I tests on residuals of best models.

| Species | Summary of Moran I's tests on residuals | | | |
| --- | --- | --- | --- | --- |
|  | Observed index | Expected index | Standard deviation | p-value |
| *Pipistellus pipistrellus* | 0.005 | -0.016 | 0.025 | 0.410 |
| *Pipistrellus kuhlii* | 0.006 | -0.016 | 0.024 | 0.370 |
| *Pipistrellus nathusii* | -7.534E-05 | -0.016 | 0.024 | 0.512 |
| *Nyctalus ssp.* | -0.030 | -0.016 | 0.024 | 0.549 |
| Species richness | -9.524E-04 | -0.016 | 0.025 | 0.545 |

Table S4.8. Number of bat passes, proportion (species bat passes/total bat passes) and occurrence (% of sites where species were recorded) of species and genera from acoustic validation results. In bold are indicate species and genera which were included in statistical analyses, others contributed to the construction of the richness dependant variable.

| Species | No. of bat passes | Proportion (%) | Occurrence (%) |
| --- | --- | --- | --- |
| *Myotis ssp.* | 6 | 0.45 | 7.81 |
| *Nyctalus leisleri* | 16 | 1.20 | 6.25 |
| *Nyctalus noctula* | 32 | 2.41 | 7.81 |
| ***Nyctalus ssp.*** | **54** | **3.61** | **10.94** |
| ***Pipistrellus kuhlii*** | **68** | **5.12** | **35.94** |
| ***Pipistrellus nathusii*** | **79** | **5.95** | **34.38** |
| ***Pipistrellus pipistrellus*** | **1125** | **84.71** | **67.19** |
| ***Pipistrellus ssp.*** | **1272** | **95.78** | **68.75** |
| *Plecotus ssp.* | 2 | 0.15 | 3.13 |

Table S4.9. Pseudo R² for each covariable of full models calculated from generalized linear models (GLM) only including the farming system and date variables (and species for *Pipistrellus ssp.* model). R² were calculating using “1-(null deviance/residual deviance)” formula.

| Dependant variables | Independant variables | | | | | | | |
| --- | --- | --- | --- | --- | --- | --- | --- | --- |
|  | Farming system | Forest | Wetland | Urban | Hedgerow | Boundaries | Road |  |
| *Nyctalus ssp.* | **0.34** | 0.19 | 0.15 | - | 0.17 | - | - |  |
|  |  |  |  |  |  |  |  |  |
| *Pipistrellus kuhlii* | **0.32** | - | 0.20 | - | 0.27 | - | 0.20 |  |
|  |  |  |  |  |  |  |  |  |
| *Pipistrellus nathusii* | **0.28** | - | - | - | 0.19 | 0.21 | 0.22 |  |
|  |  |  |  |  |  |  |  |  |
| *Pipistrellus pipistrellus* | **0.31** | - | 0.18 | - | - | 0.16 | 0.17 |  |
|  |  |  |  |  |  |  |  |  |
| *Pipistrellus ssp.* | **0.50** | - | 0.43 | - | 0.44 | - | 0.43 |  |
|  |  |  |  |  |  |  |  |  |
| Richness | **0.33** | - | 0.17 | - | 0.19 | - | 0.12 |  |
